# Clinical Case Series of Decrease in Shear Wave Elastography Values in Ten Diabetic Dyslipidemia Patients Having NAFLD with Saroglitazar 4 mg: An Indian Experience

**DOI:** 10.1155/2020/4287075

**Published:** 2020-03-27

**Authors:** Sayak Roy

**Affiliations:** Dept. of Internal Medicine, Medica Superspeciality Hospital, Kolkata, India

## Abstract

**Background:**

Diabetes and other metabolic abnormalities including high triglycerides (TGs) are commonly seen comorbid conditions in patients having nonalcoholic fatty liver disease (NAFLD). There is no approved pharmacotherapy for NAFLD, and life-style therapy plays a major role. Saroglitazar, the world's first approved dual PPAR *α*/*γ* agonist, is approved in India for the treatment of diabetic dyslipidemia. The objective of this case series analysis was to evaluate the safety and effectiveness of saroglitazar 4 mg once daily in reducing liver stiffness in patients having diabetic dyslipidemia associated NAFLD.

**Method:**

In this retrospective case series analysis, we identified 10 patients with diabetic dyslipidemia (type 2 diabetes and triglycerides >200 mg/dL at baseline) and NAFLD who were treated with saroglitazar 4 mg once daily and the follow-up data were available for 9 months after saroglitazar treatment. At baseline, all patients were on stable antidiabetic and statin therapy. Liver stiffness was measured by using 2D shear wave elastography at baseline and at 9-month follow-up.

**Results:**

At 9-month follow-up after saroglitazar treatment, significant improvement was observed in shear wave velocity (SWV) and serum transaminases levels. Serum TG level was significantly reduced after 9-month treatment with saroglitazar. No major adverse event was reported.

**Conclusion:**

In this case series of 10 patients with diabetic dyslipidemia and NAFLD, saroglitazar improved liver stiffness along with reduction observed in liver enzymes and TG values. Long-term randomized controlled clinical trial is required to further establish the safety and efficacy of saroglitazar in treatment of NAFLD.

## 1. Background

Nonalcoholic fatty liver disease (NAFLD) is projected to become the leading cause of liver transplantation [1]. Insulin resistance has been considered as a core pathophysiological factor in NAFLD [2]. There has been a strong association observed between NAFLD and type 2 diabetes mellitus (T2DM), and the studies have shown that >70% of patients with T2DM will have NAFLD [3]. In NAFLD, the risk of liver-related mortality increases exponentially with increase in fibrosis stage [4]. The recommended gold standard in the diagnosis and staging of liver fibrosis is liver biopsy, but it is invasive. The use of shear wave elastography (SWE) in the diagnosis and staging of liver fibrosis has been increasing. The SWE method uses the values of acoustically produced SW propagation speeds in the liver tissue then estimates the liver stiffness [5].

Currently, there is no FDA approved pharmacotherapy for T2DM patients with associated NAFLD/NASH. Saroglitazar is the first approved dual PPAR *α*/*γ* agonist, approved in India for the treatment of diabetic dyslipidemia [6]. Here we retrospectively analyse the case series of 10 type 2 diabetic patients who also had dyslipidemia with NAFLD and were treated with saroglitazar 4 mg once daily for the period of 9 months.

## 2. Materials and Methods

We retrospectively identified the database of patients who had been prescribed saroglitazar 4 mg (Lipaglyn™, from Cadila Healthcare Ltd, containing saroglitazar magnesium) for the treatment of diabetic dyslipidemia from author's clinic. We selected patients on the following criteria for this case series analysis:

1. Patients with type 2 DM having TG > 200 mg/dL whose baseline and 9-month posttreatment parameters were available
2. Presence of NAFLD (defined sonographycally and from history) and underwent shear wave elastography for liver stiffness assessment
3. Treated with saroglitazar magnesium 4 mg once daily
4. No use of GLP1 analogue or SGLT2I or pioglitazone in the population during the assessment period or 3 months prior to assessment
5. No history of liver injury by any chemicals or drugs during the 9 months of assessment period
6. Hepatitis B and Hepatitis C negative status
7. On stable therapy for any chronic disease for >6 months prior to recruitment

Shear wave elastography machine used for liver stiffness assessment was Philips Affinity 70® using 2 dimensional imaging. This technology measures shear wave velocity (SWV) using acoustic radiation force impulse (ARFI) (Table 1).

### 2.1. Statistical Analysis Applied

The statistical analysis was done using GraphPad insta online version® (https://www.graphpad.com/quickcalcs/ttest1/) and results were analysed applying paired *t* test. A *P* value of <0.05 was taken as statistically significant.

## 3. Results

We analysed a total of 10 patients (male : female 7 : 3) with mean age of 59.3 years with average duration of diabetes 8.8 years (Table 2). The mean baseline HbA1c and TG values were 7.8% and 298.2 mg/dL, respectively (Table 3). All these patients were already on stable dose of statin therapy and antidiabetic therapy at baseline. They all were prescribed saroglitazar 4 mg once daily. The baseline and 9-month follow-up data were analysed after adding saroglitazar therapy.

**Figure 1:**
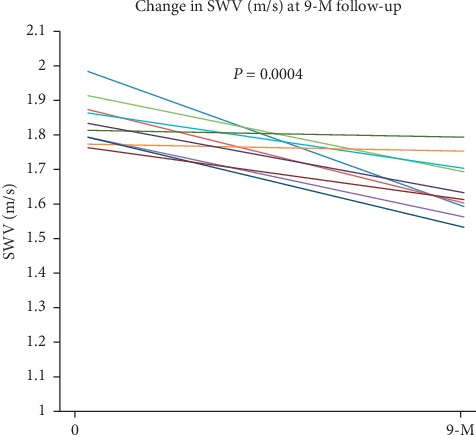
SWV of 10 patients at baseline and after 9-month treatment with saroglitazar.

The mean baseline SWV value 1.837 ± 0.0691 m/s was reduced significantly to 1.645 ± 0.0844 m/s at 9-month follow-up after saroglitazar treatment (Table 3; Figure 1). At 9-month follow-up after saroglitazar, the HbA1c level was significantly reduced from 7.8 ± 0.343% to 6.9 ± 0.33% (*P* < 0.0001). Serum TG was also significantly reduced from 298.2 ± 35.75 mg/dL to 202.4 ± 13.19 mg/dL (*P* < 0.0001) at 9-month follow-up. Liver enzymes, ALT, and AST were also reduced significantly from baseline at 9-month follow-up after saroglitazar treatment (Table 3).

## 4. Discussion

Obesity, insulin resistance, and other metabolic abnormalities including high TG are very closely related to NAFLD. Recently, a retrospective study showed a huge burden of liver fat score as measured by SWE in our T2DM patients having NAFLD [7]. Current guidelines recommend that the management of NAFLD should consist of treating liver disease as well as the associated metabolic comorbidities such as obesity, hyperlipidemia, insulin resistance, and T2DM [8]. Saroglitazar has been studied in India in patients with diabetic dyslipidemia during phase III RCTs [9]. Saroglitazar significantly improves insulin resistance by its action on PPAR *γ* receptor [10]. This study showed significant improvement in SWV values with additional improvements in metabolic parameters (HbA1c, TG and HDL-C) with 9-month treatment with saroglitazar. Saroglitazar also improved serum transaminases levels at 9-month treatment. In this case series, saroglitazar treatment was not associated with increase in BMI and waist circumference (Table 3) at 9-month follow-up.

### 4.1. Limitations of This Case Series

The sample size was small to deduce any proper conclusion. There was no chance of intervention in life-style changes, and hence, the differences in SWV value reduction cannot be properly attributed to the molecule per se. The robust reduction of HbA1c might be attributed to the change in other oral hypoglycemic drugs whose doses were increased to meet the HbA1c target of 7%.

### 4.2. Summary

In this case series, 9-month treatment with saroglitazar showed significant reduction in SWV values along with improvement in metabolic parameters (blood glucose, TG, and HDL-C levels) without causing weight gain in Indian type 2 diabetic dyslipidemia patients with NAFLD. This is a retrospective case series analysis of only 10 patients, and a large randomized controlled clinical trial is needed to establish the safety and efficacy of saroglitazar in patients with NAFLD.

## Figures and Tables

**Table 1:** ARFI (acoustic radiation force impulse) values for liver stiffness by SWE.

ARFI grade	ARFI values (m/sec)
Normal	1.0–1.5
Mild fibrosis	1.5–1.75
Moderate fibrosis	1.75–2.1
Severe fibrosis	>2.1

**Table 2:** Baseline characteristics.

Sr. No. of patient	Age (yrs)	Sex	BMI (kg/m^2^)	Waist circum (inches)	Duration of T2DM (yrs)	HbA1c %	TG (mg/dL)	ALT (U/L)	AST (U/L)	HDL-C (mg/dL)	Systolic blood pressure (mmHg)
1	52	M	26.5	40.5	7	7.9	330	68	66	35	132
2	55	M	27	41	10	8.1	280	88	43	43.2	138
3	63	M	29.3	41.1	11	8.3	311	54	42	40	142
4	64	M	25.3	40	8	7.9	270	67	39	37	134
5	53	M	22.4	39.8	7	7.2	298	71	39	45	136
6	57	F	21.5	35	8	7.4	252	81	43	48	128
7	61	F	27.6	34.3	9	7.8	307	41	28	46.2	126
8	62	F	22.5	35.1	8	8.1	263	69	56	45.5	142
9	67	M	28.8	33.2	10	7.8	297	68	40	37	138
10	59	M	21.2	36	10	7.5	374	40	38	31	144

**Table 3:** Change in biochemical/clinical parameters and SWV values at baseline and after 9-month treatment with Saroglitazar 4 mg once daily.

	Baseline	9 months	Change from baseline	*P* value
Waist circumference (inches)	37.6 ± 3.138	37.51 ± 3.123	−0.09	0.54
BMI (kg/m^2^)	25.21 ± 3.07	24.81 ± 2.95	−0.4	0.046
FBG (mg/dL)	139.5 ± 15.67	106.5 ± 14.94	−33	<0.0001
HbA1c (%)	7.8 ± 0.343	6.9 ± 0.33	−0.9	<0.0001
TG (mg/dL)	298.2 ± 35.75	202.4 ± 13.19	−95.8	<0.0001
HDL-C (mg/dL)	40.79 ± 5.63	45.2 ± 4.36	4.4	0.0007
ALT (U/L)	64.7 ± 15.56	46.2 ± 12.6	−18.5	0.0058
AST (U/L)	43.4 ± 10.48	35.4 ± 6.59	−8	0.0321
SWV (M/S)	1.837 ± 0.0691	1.645 ± 0.0844	−0.192	0.0004
SBP (mmHg)	136 ± 6.04	134.6 ± 7.24	−1.4	0.0445

*n* = 10; data are mean ± SD; paired “*t*” test.
